# From papers to practices: district level priority setting processes and criteria for family planning, maternal, newborn and child health interventions in Tanzania

**DOI:** 10.1186/1472-6874-11-46

**Published:** 2011-10-21

**Authors:** Dereck Chitama, Rob Baltussen, Evert Ketting, Switbert Kamazima, Anna Nswilla, Phares GM Mujinja

**Affiliations:** 1 Nijmegen International Center for Health Systems Research and Education (NICHE), Radboud University, Nijmegen, The Netherlands; 2 School of Public Health and Social Sciences (SPHSS), Muhimbili University of Health and Allied Sciences, Tanzania; 3 Ministry of Health and Social Welfare (MoHSW), Tanzania

## Abstract

**Background:**

Successful priority setting is increasingly known to be an important aspect in achieving better family planning, maternal, newborn and child health (FMNCH) outcomes in developing countries. However, far too little attention has been paid to capturing and analysing the priority setting processes and criteria for FMNCH at district level. This paper seeks to capture and analyse the priority setting processes and criteria for FMNCH at district level in Tanzania. Specifically, we assess the FMNCH actor's engagement and understanding, the criteria used in decision making and the way criteria are identified, the information or evidence and tools used to prioritize FMNCH interventions at district level in Tanzania.

**Methods:**

We conducted an exploratory study mixing both qualitative and quantitative methods to capture and analyse the priority setting for FMNCH at district level, and identify the criteria for priority setting. We purposively sampled the participants to be included in the study. We collected the data using the nominal group technique (NGT), in-depth interviews (IDIs) with key informants and documentary review. We analysed the collected data using both content analysis for qualitative data and correlation analysis for quantitative data.

**Results:**

We found a number of shortfalls in the district's priority setting processes and criteria which may lead to inefficient and unfair priority setting decisions in FMNCH. In addition, participants identified the priority setting criteria and established the perceived relative importance of the identified criteria. However, we noted differences exist in judging the relative importance attached to the criteria by different stakeholders in the districts.

**Conclusions:**

In Tanzania, FMNCH contents in both general development policies and sector policies are well articulated. However, the current priority setting process for FMNCH at district levels are wanting in several aspects rendering the priority setting process for FMNCH inefficient and unfair (or unsuccessful). To improve district level priority setting process for the FMNCH interventions, we recommend a fundamental revision of the current FMNCH interventions priority setting process. The improvement strategy should utilize rigorous research methods combining both normative and empirical methods to further analyze and correct past problems at the same time use the good practices to improve the current priority setting process for FMNCH interventions. The suggested improvements might give room for efficient and fair (or successful) priority setting process for FMNCH interventions.

## Background

The importance of reproductive health (RH- see appendix, footnote 1) is well recognized [1-3] and articulated in the Programme of Action of the International Conference on Population and Development (ICPD) in 1994 [4]. Also, in 2005 the United Nations world summit emphasized the role of universal access to reproductive health (RH) in achieving the MDGs [5]. Yet, more than fifteen years after the ICPD and more than five years after the summit, the progress to improve access to good quality RH services has been stalled in most of the Sub-Saharan countries [6]. Less attention to RH services in the national development policies and inadequate resources are often cited as reasons for poor RH services and eventually poor RH outcomes [[2,3,6], and [7]]. As a result, there has been considerable interest in improving national policies on RH [8], and requests for increased investment in RH interventions. Until recently, nearly all sub-Saharan countries had policies emphasizing preventing and treating reproductive health problems [9].

Although the progress to achieve universal access to RH services continues to be slow and the necessary resources for RH both domestic and international continue to be scarce, little attention has been directed toward understanding and analyzing the priority setting processes and criteria in RH service delivery. Walt and Gilson stress the need to understand the processes in explaining why desired policy outcomes fail to emerge [10]. On the contrary, Smith argues that having good policy content does not automatically contribute to better outcomes, but rather the policy implementation process has greater impact on the policy outcomes [11]. We extend both Walt and Gilson, and Smith's arguments to priority setting in RH by arguing that good policy content alone, even when made correctly, will not by itself produce the desired RH outcomes, but rather successful priority setting at the policy implementation level will be helpful in achieving the desired RH outcomes.

To date there has been no or little agreement on what constitute a successful priority setting. Thus, different disciplines offer their own perspective on how priority setting needs to be done through values such as efficiency, equity and justice [12]. In this paper we define successful priority setting as one done efficiently and fairly [13]. We adopt Gibson et al definition of efficient priority setting as one which improves institutional capacity for allocating resources and making priority setting decisions and providing worthwhile return on the time invested to set priorities [13]. Also, a fair priority setting as one in which all stakeholders felt engaged in the priority setting process, making justifiable and reasonable decisions and both losers and winners felt fairly treated [13]. However, the question remains on understanding whether the RH interventions priority setting process is efficient and fair? This calls for the need for capturing and analyzing the current RH priority setting mechanisms in sub-Saharan countries. Greater insight into existing priority setting processes s and criteria could improve the way in which institutions set priorities and hence contribute to the achievement of desired RH outcomes within family planning, maternal, newborn and child health (FMNCH).

In Tanzania, FMNCH matters are well articulated in the national policies [14-16]. In addition, family planning is defined as a health intervention and not as a demographic intervention [17], such that, the family planning services are integrated within FMNCH issues coordinated by the reproductive and child health section (RCHS) in the Ministry of Health and Social Welfare (MoHSW). At the district level, family planning services are provided within an integrated clinic structure designed for FMNCH services under the district RCHS.

District councils act as national policy implementing agencies and are required to identify the priorities and plan how the resources allocated for health will be spent to address local health needs [18]. The council health management teams (CHMTs) and council health planning teams (CHPTs) are responsible for the identification of the priority interventions to be included in the comprehensive council health plan (CCHP) [19]. Nevertheless, there are concerns that the CCHP process does not employ priority setting mechanisms suited for recognising the need and priorities of FMNCH interventions [20]. As a result, FMNCH's important interventions are often overlooked leading to poor coverage of RCH services.

Against this background this paper captures and analyses the priority setting processes and criteria for FMNCH at district level in Tanzania. Specifically, the paper aims at assessing the FMNCH actor's engagement and understanding, the criteria used in decision making and the way criteria are identified, and the information or evidence and tools used to prioritize FMNCH interventions at district level in Tanzania.

### The context

#### FMNCH status

The FMNCH status both at national and regional levels are indicated in Table 1. The table indicates that in Mwanza region FMNCH status indicators are worse than the national average. Given the current FMNCH status indicators, there is much concern if Mwanza and Tanzania in general is going to achieve universal access to reproductive health as stipulated in the Millennium Development Goals (MDGs)

**Table 1:** Social -economic and demographic characteristics of the study area

Indicators	National level	Mwanza Region	Source of data
Population size	34.4 million	2.9 million	(NBS, 2006)
Population growth rate	2.90%	3.2%	(NBS, 2010, URT., 2006)
Total Fertility rate	5.4	5.7%	(NBS, 2010, URT., 2006)
Contraceptive prevalence rate(All method)	34%	15%	(NBS, 2010)
Contraceptive prevalence rate(Modern method)	27%	12%	(NBS, 2010)
Maternal Mortality ratio	454/100000	No data	(NBS, 2010)
Neonatal Mortality rate	26/1000	55/1000	(NBS, 2010)
			
Under five Mortality ratio	81/1000	80/1000	(NBS, 2010)
Income poverty	33.30%	37.6%	(NBS, 2009)
Food Poverty	16.60%	18.4%	(NBS, 2009)
Unmet need for family planning	21.6%	26.7%	(NBS, 2005, Keogh et al., 2009)

### The district health system planning process

District councils prepare the CCHP guided by the CCHP guideline and PlanRep (See appendix, footnote 2) Software [19]. The CHMT is obliged to solicit priority interventions from the district hospital, health centres, dispensaries, community, district RCHS and other sections to be accommodated in the CCHP [19]. The comprehensive council health plan guideline requires each identified priority intervention to be selected from the national essential health package (NEHP) based on the health problem magnitude, severity, capacity of implementation and cost. The CCHP is prepared by the council health planning team, comprising of the health management team, district planning officer, representatives from the private sector, NGOs, FBOs, community development department and CHMT co-opted members.

**Figure 1:**
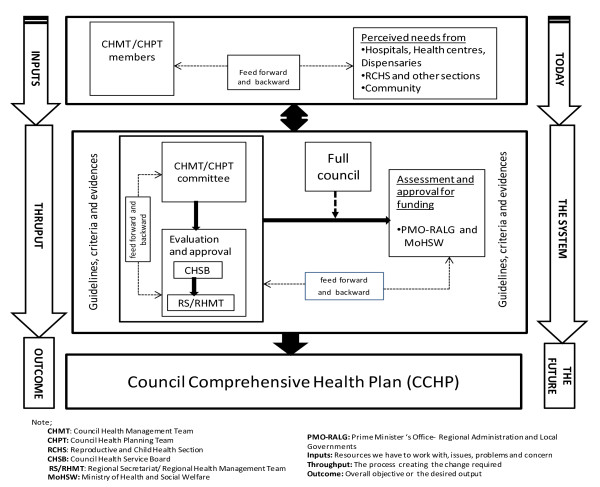
**Figure 1 shows the district council health system planning process comprising of three phases; the input, thruput and outcome phases.** The input phase involves the forth and back consultative process between the CHMT and beneficiaries in soliciting priority interventions to be considered in the CCHP. The thruput phase involves the prioritization process and approval by various authority bodies within and outside the district council. The prioritization and approval process is guided by guidelines, criteria and evidences. The outcome phase involves the approved CCHP document ready for funding and implementation.

The CCHP needs to be approved by the Council Health Service Board (CHSB) before it is sent to the Regional Secretariat (RS) for checking as to whether the CCHP conforms to the guideline. After RS checking, the CCHP and its budget are passed on to the Full council, the highest political body in the council, for final approval. After approval of the Full council, the CCHP is sent out to the MoHSW and PMO-RALG, through the RS for final checking and approval for funding. Figure 1 summarises the district health planning process in Tanzania.

## Methods

We used an exploratory study mixing both qualitative and quantitative methods to capture and analyse the priority setting for FMNCH at district level, and identify the criteria for priority setting. We purposively sampled participants from the district's RCHS staffs, CHMT, CHPT, regional and district RCHS coordinators, because our sampling strategy was conceptually driven by the research questions from the outset. All RCHS's staffs, CHMT and CHPT were included in the research to avoid within case selection biases. Also, we made random sampling of participants from the general population groups (GPGs).

### Study area

We undertook the study in Mwanza region in Tanzania in 2010. Mwanza Region lies in the northern part of Tanzania, located between latitude 10° 30' and 30° south of the Equator neighbouring Lake Victoria. At the time of this study, Mwanza region had eight districts. We randomly selected three districts councils namely, Magu, Kwimba and Misungwi districts for inclusion in the study.

### Data collection

We collected the data using a nominal group technique (NGT) also called consensus method [21,22], in-depth interviews (IDIs) with key informants and documentary review. We conducted nine NGT discussions involving 12 to 24 participants in each discussion session. Participants in the NTG included the district RCHS staffs, CHMT, CCHPT and the GPG representatives. The NGT discussions involved the following steps:

1. The session moderator presented to the participants with an initial statement of the topic to be discussed. The statements included "Strengths and weaknesses of priority setting for FMNCH in the council comprehensive health planning process". Explanations were given as to what priority setting means and its objectives. Once participants understood the topic, the discussion started.

2. The session moderator directed the participants to reflect individually on the topic and to record their personal responses in a notebook or on a piece of paper.

3. The session moderator collected the individual responses and the responses were written on the flipcharts in a concise and complete manner.

4. The session moderator initiated the discussion to consolidate and review the complete set of ideas. In addition, the discussion was broadened to identify and discuss priority setting criteria. At this stage, all flip-charts were posted so that all ideas were visible. Also, all the discussions were audio taped to ensure all discussed points were captured.

5. Compilation of the results and the ranking of the identified priority setting criteria were done to establish the perceived importance of each criterion.

Also, we conducted in-depth interviews with the District Medical Officers (DMOs), District RCHS coordinators and the Regional RCHS coordinator. In addition, we reviewed the CCHP guideline and the CCHP.

### Data analysis

We analysed the collected data using both the content analysis for analysing qualitative data and correlation analysis for quantitative data. The content analysis involved iterative and interrelated steps which included transcribing the information, coding, data reduction, data display and interpretation of themes [23]. We analysed qualitative data as follows; first, we transcribed the recorded information, and we read and re-read the data repeatedly to acquire sense of the overall data. Secondly, we coded the data by bringing together similar ideas into identified categories under the priority setting processes. Third, we did data reduction by filtering the information focusing on the most essential concepts and their relationship with the identified categories. Lastly, the data captured were analysed under various themes by identifying variations and similarities in responses.

We used ranking method to establish the perceived importance of the priority setting criteria identified in the NGT discussions as follows; first, the groups identified priority setting criteria and rank ordered the identified criteria during NGT discussions. The groups used rank 1 to denote the highest important criteria, rank 2 second important criteria and so on. Rank 0 means the criterion was not identified by the group as important. Second, we used nominal approach to calculate scores where the highest ranked criterion received a score of 15, second criterion in importance received a score of 14 and so on. The score of 15 was the highest score given to the first ranked criterion. The scores were then aggregated across all groups to provide a summary index of criteria importance. Thirdly, we used the aggregated scores to rank the identified criteria in terms of their approximate quantitative order of importance. Lastly, we tested whether there are similarities in opinions on judging the criteria between the groups using correlation coefficients. For the interpretation purpose, we arbitrarily set the correction coefficient cut offs as indicated in Table 2.

**Table 2:** Correlation coefficient cut offs

Correlation coefficient cut-offs	Explanations
1.0 to 0.7	Strong positive association in opinion
0.7 to 0.5	Modest positive association in opinion
0.5 to 0.2	Weak positive association in opinion
0.2 to -0.2	Little or no association in opinion
-0.2 to -0.5	Weak negative association in opinion
-0.5 to -0.7	Modest negative association in opinion
-0.7 to -1.0	Strong negative association in opinion

### Research ethics and permission

We obtained the ethical clearance to conduct the study from the Muhimbili University of Health and Allied Sciences (MUHAS) Ethical Review Board. Permission to collect data was granted by both Tanzania Commission for Science and Technology (COSTECH), regional and district authorities.

We sought verbal consent from prospective informants after explaining the objectives of the study. Also, we informed informants of the right to withdraw from the study at any time they wished without any consequences. We sought and obtained special permission from the participants to audio-record all IDIs and NGT in Kiswahili.

We assured participants of the confidentiality of the data/records. Thus, we promised to keep all the study data/records, including any codes in a secure location at MUHAS. However, we informed the participants that although the researchers will take every precaution to maintain confidentiality of the data/records, the nature of NTG does not preclude other participants from revealing what has been said by other NTG participants during the discussion. Therefore, we reminded participants to respect the privacy of their fellow participants and not reveal what was said by others participants during the discussions.

## Results

The FMNCH priority setting analysis at the district level generated a variety of issues. For the purpose of this paper, we arbitrary categorize the emerged issues into the actors involved in priority setting, the priority setting process, external influences and identified criteria. We exemplify the results by including supporting quotes from IDIs, NGT discussion and document review.

### Actors involved in the district health priority setting

We define actors as all the officials at the district level playing a relatively significant role in the district health priority setting process. Actors are therefore categorized based on team composition, skills and knowledge in priority setting.

### The Council Health Planning Team (CHPT) and Council Health Management Team (CHMT) composition

Team composition referred to CHMT and CHPT members. The results show that the district RCHS coordinators hardly participate in the district health planning and priority setting processes. In addition, the district RCHS are neither represented in the CHPT nor the CHMT. It was further reported that some of the important health system sections are also not involved in priority setting and writing of the plan to an extent that some of the important information that would guide priority setting are not utilised. This was exemplified in the NGT discussions as follows:

"The proposed structure in the Council Comprehensive Health Plan (CCHP) guidelines is very good, but its translation into implementation has a problem. Just by looking at the Council Health Planning Team (CHPT) composition, you find that a team that sits to prioritize in the CCHP has no representatives from other crucial sections. Therefore, planning team just guesses the statistics and needs of the unrepresented sections. For instance, the Reproductive and Child Health Services (RCHS) coordinators are not represented in the Council Health Management Team (CHMT) and CHPT, nonetheless RCHS have a very big role to play in improving health status in the district, and so many things are needed to improve service and coverage" (NGT, 2010)

District RCHS coordinators are not permanent members of the CHMT and CCHP team. Therefore, district RCHS have to send their proposed plan to CHMT for inclusion in the CCHP. However, RCHS coordinators noted that more often important interventions are left out during prioritization process. People involved in the prioritization processes would give priority to interventions from their own departments or sections. One key informant reported:

"RCHS coordinators prepare plans and send them to Council Health Management Team for inclusion in the CCHP. The Council Health Management Team goes through the proposed plan from RCHS. Depending on how much money is available, some of the FMNCH interventions can be included and others will not. Very often, FMNCH interventions we think are important are not included in the final Council Comprehensive Health Plan (CCHP). In most cases those core participants in the CCHP preparations are biased toward interventions from their own sections"(IDIs, 2010)

### The priority setting skills and knowledge

We defined skills and knowledge as the learned capacity to carry out priority setting and the general understanding of FMNCH interventions. We found priority setting and resource allocation skills were very low at all levels starting with the district RCHS and district health system levels. Both district RCHSs and CHPTs acknowledged the use of intuitive experiences in prioritizing interventions. In addition, CHPT members' knowledge about FMNCH interventions was found to be limited. For example in the NGT discussions it was revealed that:

"We have little skills in the whole question of preparing plans. Majority of us have different primary professions skills background which are different from skills needed for preparing district health plans. Therefore we just do the planning by intuitive experience. We are lacking training and support, both technically and supervisory during the identification of health priorities in general and for FMNCH" (NGT, 2010)

This was echoed during another NTG discussion which revealed that the technical knowhow of preparing CCHP was weak:

"The technical knowhow of preparing plans is weak among CHPT members, we only use experience. Some of us are unknowledgeable with FMNCH interventions."(NGT, 2010)

### Priority setting approach

We defined priority setting approach as the methods applied in determining the district health priority list. Practically, the results show that the priority setting processes at district level are top down and incremental in nature. The CHMT rarely gets contributions from lower levels in the priority setting and resource allocation process. This was revealed by some participants in the NGT discussions revealed as follows:

"Sometimes in the CHMT pre-planning meetings we assume the needs for FMNCH in the district will be this and that. We assume we are making correct guesses. As a result the identified needs are not real because they do not originate from the district RCHS" (NGT, 2010)

Another key informant in the in-depth interview highlighted the use of an incremental approach in determining the district priority needs.

"*We use the previous year's plan as a base for next year's projections. We just look on previous year's target, what we planned and what did we achieve; then we predict what is required" (NGT, 2010)*

### Priority setting criteria

Theoretically, prior to examining options for funding, the planning teams must determine a set of decision making criteria on which the priority setting will be based. The results show that in all districts priority setting processes, none had explicitly stated criteria nor applied the criteria proposed in the guideline. This was further revealed in the NTG discussion that the CHMT arbitrarily agrees on what is an important and what is not an important intervention for funding.

"We arbitrarily agree on what interventions are important and not important. Sometimes it happens that those interventions which are important are left out and those which are not important are prioritized for funding" (NGT, 2010)

In addition, the CCHP guideline review shows that the essential health package, burden of disease profile, council performance indicators, National Strategy for Growth and Poverty Reduction (NSGPR) priorities and the MDGs are listed as criteria for priority setting. However, the mentioned criteria are not weighted to show their relative importance. The CCHP guideline read as follows:

"In Tanzania the following tools are well developed to give guidance for priority setting of primary health problem; essential health package, burden of disease profile, council performance indicators, NSGPR and MDGs" (MoHSW, 2007a)

### The use of evidences in priority setting

Theoretically, different information, particularly relevant locally available information from different sources is required during the priority setting process. We found, there is a practical problem of the amount and quality of information needed in the prioritization process. In most cases, FMNCH information used in priority setting was incomplete or inaccurate. The NGT participants had different explanations for the incompleteness and inaccuracy of the FMNCH information; as one participant narrated that:

"We are lacking credible statistics/data that are related to the real situation of district RCHS needs. Therefore, we may be preparing CCHP using wrong FMNCH data (...). In collecting data, we use the MTUHA ^(^Appendix, footnote 3) system. Data quality and accuracy in MTUHA are not sufficiently assured and there is late reporting of health information from one level to another. We also get other demographic data from District planning offices" (NTG, 2010)

Another participant added that:

"We are lacking accurate data showing the real FMNCH situation in the district (...) absence of data frustrates a lot (...) So it is difficult to know which FMNCH problems are more important" (NGT,2010)

Another participant commented that:

"Correct data are not available in the district's RCHS, or they don't exist at all, if available don't show the real FMNCH picture. The priorities which are shown in the plan may not be real, because we lack correct FMNCH data" (NTG, 2010)

### The priority setting tools

**Figure 2:**
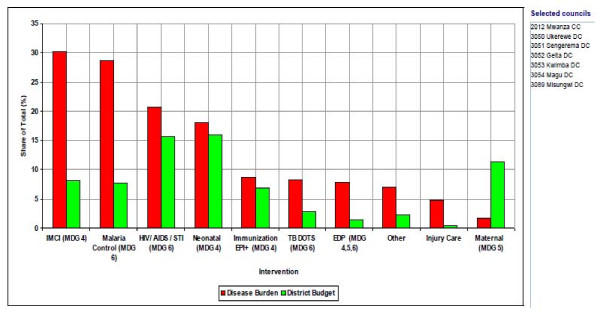
Figure 2 is an extract from the district health account (HDA) tool used in the prioritization process. The extract maps out the summary of the previous year priority intervention’s share of expenditure and its corresponding share of the BOD to guide the current year’s prioritization process. The assumption is the intervention with high BOD deserves high priority in resource allocation and vice versa. However, this assumption is not the case in some district council health system planning process.

The Prime Minister's Office Regional Administrations and Local Governments (PMO-RALG- Appendix, footnote 4) in collaboration with Ministry of Finance and Economic Affairs (MOFEA) designed software to assist local authorities in planning, budgeting, projecting revenue, track funds received, physical implementation and expenditure. The software incorporates the district health account (DHA) tool under MOHSW which is required for priority setting, targeting resources to interventions addressing the largest share of burden of disease (BOD) and producing graphics and summaries of CCHP (MoHSW, 2007a). Despite neonatal, maternal and Integrated Management of Childhood Illness (IMCI) interventions having a reasonable share of expenditures, the corresponding FMNCH outcome indicators are worse than the national averages. Figure 2 shows the BOD against expenditure share in Mwanza region.

### External influences

We defined external influences as directives which were out of control of the district priority setting process coming from outside the district health planning process requirements. While we acknowledge the broader context of external influences, it was not the focus of this paper to broadly examine the role of the external influences on priority setting at district level. We, therefore, focus on the donor and central government influence only. The results show that both the donors and government influence priority setting processes at district level.

### Donor influence

District councils receive funds from different sources including the donors through the Health Basket Funding mechanism. Results show that in most cases the donated funds come with another string of priorities attached. The donors dictate what will be spent and where. The donor priorities are sometime not in favor of FMNCH interventions at the districts. Participants in the NGT discussion revealed that some of the donors have their priorities that are different from the district's priorities.

"We prepare plans basing on our guidelines, but sometime it happens that some of the donors/funders bring money with their own attached objectives/priorities. For example, the district priority could be constructing a district RCHS building/house, but funders say they don't want us to build a house, they prefer just renovation. This results in doing what is not really needed in our districts" (NGT, 2010)

### Government instructions

Results show that in some cases, the districts have to align their priorities to the government directives which may not be necessarily on the districts priority list. This was revealed by participants in the NGT discussion as follows:

"Sometimes we get instructions from the MoHSW. For example this year we were directed to buy delivery kits for every health centre (...) we are wondering who had identified the delivery kits a top priority on FMNCH needs." (NGT, 2010)

Another NGT discussion participant added:

"For instance we are instructed that the issue of traditional medicines should be included in the plans, but in our district the issue of traditional medicines does not constitute a major problem" (NGT 2010)

### The identification priority setting criteria

A total of 15 priority setting criteria were identified from the NGT discussion (Table 3). The local burden of the problem was found to be the most important criterion identified by the district's RCHS and the GPG. The CHPTs considered prevention as most important criterion followed by intervention cost criterion. Generally, there was much diversity of the perceived importance of the identified criteria between district RCHS, CHPT and the GPG. Table 3 shows the order of importance of the identified criteria by district RCHS, CHPT and GPG in all the three districts combined. Table 3 shows that there is very little correspondence in priority criteria between the three different groups. Surprisingly, none of the three groups mentioned international goals as a criterion.

**Table 3:** Order of importance of the identified criteria by different groups of actors

Criteria	Reproductive and Child Health Sections (RCHS)	Council Comprehensive Health Plan Teams (CCHPT)	General Population Groups (GPG)
Local Burden of the problem	1	8	1
Effectiveness	2	4	2
Capacity(HR,Equipements etc	3	7	8
Number of beneficiaries	4	3	4
Preventive	5	1	3
Society preference	6	6	5
Cost	7	2	0
National policy priority	7	5	0
Poverty reduction	9	11	6
Target area(Rural vs Urban)	10	0	0
Previous year's target	11	0	0
Positive externality	12	12	0
International goals	0	0	0
Political support	0	10	0
Vurnerable groups	0	9	7

### Correlation analysis results

We conducted correlation analysis to determine whether there is association in opinions on judging the priority setting criteria between the groups in the districts and in all groups in general. We found a positive association of opinion on the importance of various priority setting criteria in the GPG between the districts (Table 4).

**Table 4:** General population group's correlation analysis

	District a	District b	District c
District a	1.0000		
District b	0.3959	1.0000	
District c	0.5327	0.6809	1.0000

We found two out of three RCHSs in the districts had little or no association of opinion on the importance of various priority setting criteria and the remaining had weak positive association (Tables 5).

**Table 5:** Reproductive and child health section's correlation analysis

	District a	District b	District c
**District a**	1.0000		
**District b**	0.2696	1.0000	
**District c**	0.0940	0.7956	1.0000

We found two out of three CHPTs in the districts had little or no association of opinion on the importance of various priority setting criteria and the remaining had weak positive association (Tables 6).

**Table 6:** Council health planning team's correlation analysis

	District a	District b	District c
District a	1.0000		
District b	0.5811	1.0000	
District c	-0.0972	0.3542	1.0000

We found the GPG and CHPT, and CHPT and district RCHS had little or no association of opinion on the importance of various priority setting criteria while the GPG and the CHPT had a weak positive association (Table 7).

**Table 7:** All districts combined scored ranking correlation analysis

	GPG	RCHS	CHPT
GPG	1.0000		
RCHS	-0.2683	1.0000	
CHPT	0.3468	-0.1014	1.0000

## Discussion

The primary purpose of this paper was to capture and analyze the priority setting processes and criteria for FMNCH at district level. Our findings suggest there are shortfalls in district health priority setting processes and criteria, which can lead to inefficient and unfair priority setting decisions. We found the district RCHS coordinators responsible for implementation of FMNCH interventions in the districts are not engaged in the district health planning teams, the priority setting processes are ad hoc and implicit, the use of incomplete and inaccurate FMNCH information during prioritization, low skill and knowledge of the priority setting team members on FMNCH and priority setting, and the bargaining nature of the prioritization process due to lack of criteria. Given these shortfalls, there is little chance FMNCH interventions prioritization will be efficient and fair. Therefore, the scope and breadth of FMNCH services to be delivered at district level will remain limited and likely to lead to poor FMNCH outcomes. Daniels and Sabin noted that under such conditions procedural fairness in setting priorities is essential for reaching a fair decision [24].

According to MoHSW [19], the Regional Health Management Teams (RHMTs) are supposed to provide priority setting technical support and supervision to the districts. In reality, the RHMTs rarely conduct technical and supervisory support to both district's RCHs and CHPTs despite having a seat in the CHPT. In the absence of technical and supportive supervision coupled with low understanding of priority setting due to low skill and knowledge among the RCHS and CHPT members, complete scanning of the interventions and advance warning of the possible undesirable impact will be missing. As a result, the few resources available are more likely to be allocated to interventions with a negligible improvement in FMNCH outcomes. In this context, capacity building through priority setting trainings and other activities like technical and supportive supervisions, mentoring and coaching are very much needed.

In the same vein, the issue of low skills and knowledge of the priority setting team members was reflected in the capacity to use the available priority setting tools. Districts are provided with the district health account (DHA) tool. The DHA is required for priority setting to target resources to interventions addressing the largest share of burden of disease (BOD). However, we found the opposite scenario. We found interventions addressing the lower BOD were allocated a reasonable share of expenditures. Interestingly, the corresponding outcome indicators for these interventions are worse than the national averages. There is no direct explanation for this anomaly. However, indirect explanations for it would be either the inaccurate information or tools available are not sufficient to address the priority setting challenges or the priority setting teams lack necessary capacity to analyze available information and proper use of the DHA. In the event of low capacity, priority setting teams tend to opt for simple solutions to complex problems resulting in less improvement of the desired outcomes.

The findings on priority setting practices and criteria are consistent with other studies done elsewhere. For instance, Maluka and others revealed that local institutional context and power asymmetries among actors have great influence on priority setting process at district level [25]. Mayhew and Adjei found traditional priority-setting tools do not adequately reflect the long-term benefits of preventive interventions such as family planning, and the priority-setting processes were exclusive of RH specialists [26]. Consequently, the RH goals were not reflected in the health priority list in Ghana. Kapiriri and Martin noted priority setting in developing countries is fraught with uncertainties due to lack of credible information, weak institutions and unclear priority setting processes [27]. Ham and Coutler (2001), and WHO (2004) propose solutions for improving the priority setting processes. While WHO proposes improving information for priority setting in efforts to build and strengthening the RH capacity[7], Ham and Coutler propose to strengthen institutional processes in which priority setting decisions are taken [28].

Also, this paper aimed at exploring the criteria used in the priority setting process and further explores their relative importance at the district level. Despite many criteria for priority setting being proposed and debated [29-32] and the CCHP guideline in place, we found no explicit criteria are used to prioritize different interventions both at district RCHS and health system levels. Participants of the planning teams arbitrarily agree on what is an important and what is not an important intervention. Thus, priority setting decisions are always based on reasons that were not grounded in explicit criteria. In the absence of explicit criteria, resources will always be allocated to interventions dictated by influential members of the decision making panels especially when power differences exist in the priority setting team. Gibson et al noted that power differences may have the effect in determining the reasons that inform priority setting and hence undermining the overall process efficiency and fairness [13]. Since district RCHS coordinators are not represented in the planning team or co-opted, it is expected that there will be inefficient and unfair priority setting in FMNCH interventions.

We found the CCHP guideline proposing a number of criteria to be used in preparation of the CCHP. The criteria proposed included essential health package, burden of disease profile, and council performance, NSGPR and MDGs indicators. However, the proposed criteria are not weighted to reflect their relative importance nor used in the priority setting process. The absence of weights on the proposed criteria turns the priority setting process into intuition and bargaining practices. Thus, the priority setting teams struggle to set priorities appropriately because they lack consensus about which criteria weight should guide their priority setting decision. Under this scenario, good FMNCH interventions that give maximum benefits are likely to be left out in the district health priority list. Mitton and Donaldson argue that if criteria are not explicitly weighed, it implies equal weight across the criteria which may not be reflective of the underlying values of the priority setting team and other stakeholders [33].

We explored the opinion on the criteria and their relative importance from the general population group (GPG), the reproductive and child health services (RCHS) groups and the council health planning team (CHPT) groups. The relative importance attached to the criteria may reflect the values of the given organization or the health system, value of the staff within the organization or more broadly, the society at large guiding the decisions that must be taken. For instance, Robert et al noted that utilitarianism focuses on consequences, liberalism focuses on rights and opportunities and communalism focus on the kind of individual and communities that need to be created [34].

The groups identified a set of priority setting criteria which were more or less identical their relative importance. However, we noted the disagreement between the GPG, RCHS and CHPT exist in judging the relative importance attached to criteria by different groups (Table 3,4,5 and 6), which was interesting. We found little or no association in opinion between the RCHS, CHPT and the GPG (table 7). These findings suggest that there are competing values and criteria between district RCHS's and CCHPT's need to be considered during priority setting. The absences of consensus on the values and criteria have implications for the district's final priority setting decision and service uptake by the general population. This is because the planning teams are likely to priorities FMNCH interventions not identified by the district RCHS as important. Also, the RCHSs are likely to propose FMNCH interventions not preferred by the general population. Daniel and Sabin noted that in pluralist societies we are likely to find reasonable disagreements about principles that should govern priority setting [24]. In the absence of consensus on the principles, a fair process allows to agree on what is legitimate and fair [24]

Although we capture and analyze the FMNCH priority setting practices and criteria in three out of eight districts in the lake zones, there is no reason to believe that the insights gained from this study are not transferable to other similar settings in Tanzania. This is because the structures and organizational contexts of the district health systems in Tanzania do not greatly differ. Therefore, FMNCH priority setting stakeholders in other districts may use the findings of this study as a basis for discussing how they could enhance the efficiency and fairness in their own FMNCH priority setting process. In addition, the lessons we report fill an important gap in the literature about priority setting processes and criteria.

## Conclusions

In this context, an implicit assumption often is that once a policy has been adopted by the government, it will be implemented and the desired outcomes will be achieved. But in practice this assumption is invalid. Implementing agency's processes, especially the priority setting and resource allocation mechanisms, in practice also play an important role in achieving the expected policy's results. In Tanzania, FMNCH contents in both general development policies and sector policies are well articulated. However, the current priority setting process for FMNCH at district levels are wanting in several aspects. There are practical problems of FMNCH stakeholder's engagement, and the amount and quality of FMNCH information and planning tools used during prioritization. The priority setting processes are ad hoc and implicit, low skill and knowledge of the priority setting team members on FMNCH interventions, and the bargaining nature of the prioritization process due to lack of criteria and lack of the technical and supervisory support to priority setting team rendering the priority setting process for FMNCH inefficient and unfair (or unsuccessful).

To improve district level priority setting process for the FMNCH interventions, we recommend a fundamental revision of the current FMNCH interventions priority setting process. Specifically, we recommend FMNCH stakeholder's engagement in the priority setting process, training and availing the technical and supervisory support to priority setting teams, improvement in the use of FMNCH information and planning tools; and development of an efficient and fair priority setting process/framework. The improvement strategy should utilize rigorous research methods combining both normative and empirical methods to further analyze and correct past problems at the same time use the good practices to improve the current priority setting process for FMNCH interventions. The suggested improvements might give room for efficient and fair (or successful) priority setting process for FMNCH interventions. The efficient and fair (or successful) priority setting process will facilitate optimal coverage of Reproductive and Child Health (RCH) services, which are likely to have a positive impact on both national and international development goals on family planning, maternal, newborn and child health.

## Competing interests

The authors declare that they have no competing interests.

## Authors' contributions

DC conducted the nominal group discussions on which this paper is based, collated and analyzed in the data and drafted the manuscript. RB, EK, PGMM and SK participated in analyzing the data and commented on the earlier draft of the manuscript. AN commented on the earlier draft of the manuscript. All authors read and approved the final manuscript.

## Appendix

### Footnotes

1. The authors recognize that the definition for RH as per UN (1994) is very broad and holistic in nature. Reproductive health has thus become multidimensional and its policies and interventions now extend beyond health per se. For the purpose of this paper we limit the discussion on reproductive health outcomes as measured by indicators such as maternal, neonatal and child mortality, and contraceptive prevalence rate.

2. Abbreviation for Planning and Reporting

3. Kiswahili acronym for "Mfumo wa Taarifa za Uendeshaji wa Huduma za Afya" meaning Health Management Information System (HMIS). The system covers all health programmes and health care services. All health facilities (Government, some Private, NGOs and Parastatals organizations) use the MTUHA system (MoHSW, 2002)

4. Prime Minister's Office Regional Administrations and Local Governments

## Pre-publication history

The pre-publication history for this paper can be accessed here:

http://www.biomedcentral.com/1472-6874/11/46/prepub
